# A thin-film electrode exhibiting Nernstian pH response via a proton-coupled electron-transfer redox couple

**DOI:** 10.1080/14686996.2026.2694964

**Published:** 2026-07-07

**Authors:** Jiamin Tang, Tomoki Furukawa, Pushi Wang, Takuma Ohashi, Masaki Ishii, Jun Takeya, Katsuhiko Ariga, Yu Yamashita

**Affiliations:** a Research Center for Materials Nanoarchitectonics (MANA), National Institute for Materials Science (NIMS), Tsukuba, Japan; b Department of Advanced Materials Science, Graduate School of Frontier Sciences, The University of Tokyo, Kashiwa, Japan

**Keywords:** Proton-coupled electron transfer, pH sensor, thin-film device

## Abstract

Accurate pH monitoring in flexible thin films is essential for biochemical sensing applications. However, in conventional soft-material-based film-type pH sensors, the apparent pH response is often influenced by variations in thin-film morphology, such as swelling, microstructural disorder, and structural inhomogeneity. Here, we adopt a different strategy by confining a small-molecule proton-coupled electron-transfer (PCET) redox couple, traditionally used in homogeneous aqueous solutions, within an ion gel. A benzoquinone/hydroquinone (BQ/HQ) redox couple is incorporated into an ion-gel matrix deposited on a gold electrode and covered with a Nafion overlayer. The open-circuit potential of the Au/ion-gel interface, measured against an Ag/AgCl reference electrode, exhibits a Nernstian dependence on pH, indicating that the proton-coupled BQ/HQ redox equilibrium is preserved in the thin-film device. These results demonstrate that PCET redox couples homogeneously dissolved in ion gels provide a viable route toward compact and reliable pH-sensing films.

## IMPACT STATEMENT

This work demonstrates that reliable organic pH sensors based on proton-coupled electron transfer can be implemented as flexible thin films, with potential for biochemical sensing, multi-point measurements, and data-driven applications.

## GRAPHICAL ABSTRACT

**Figure:**
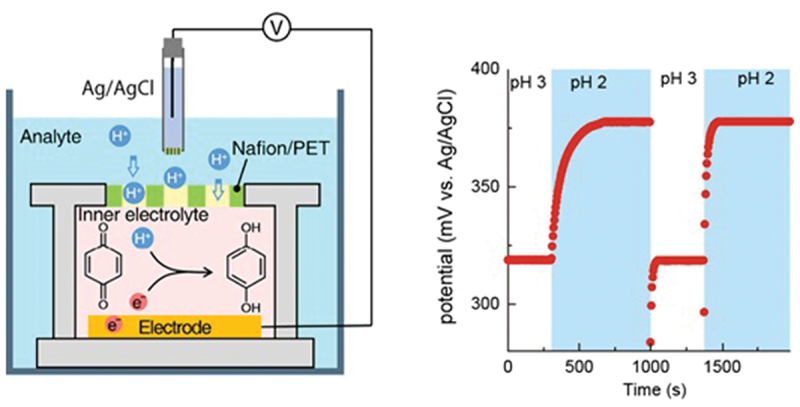

## Introduction

pH is a fundamental descriptor of aqueous chemistry that governs reaction equilibria and kinetics, corrosion and degradation of materials, biological function, and environmental water quality [1–3]. In biochemical applications in particular, pH provides a direct readout of local microenvironments and physiological states, and thus motivates continuous and miniaturized sensing in contexts such as wound-care dressings and wearable platforms for biofluids [4]. In addition, on-chip pH monitoring in microfluidic cell culture systems enables dynamic readout of extracellular acidification and metabolic activity [5].

Although glass pH electrodes and ion-sensitive field-effect transistors (ISFETs) are well-established and reliable pH-sensing technologies, both are inherently rigid and relatively bulky [6]. The glass electrode relies on a fragile glass membrane and a thick body structure, which limits mechanical flexibility and complicates integration into compact or wearable systems. ISFET devices are more easily miniaturized, but they are typically fabricated on rigid substrates and require complex packaging for stable operation [7–9]. These characteristics impose practical constraints in bioapplications, where conformability, mechanical flexibility, and integration with soft materials are often required [4]. Therefore, the development of pH sensors that can be fabricated as thin, flexible films is strongly desired for emerging applications in wearable devices, microfluidic systems, and distributed biochemical sensing [10].

Organic materials have also been explored for flexible pH sensing [11]. In particular, conducting polymers such as polyaniline have shown relatively low drift compared to other organic systems [12,13]. However, based on the reported drift rates, a deviation of 0.1 pH typically occurs within approximately 10 h to a few days. Further improvements in long-term stability and reliability are therefore required to expand their applicability. However, organic thin-film sensing materials generally suffer from issues such as structural inhomogeneity, swelling, and local variations in composition, which can lead to device-to-device variability, signal drift, and deviations from ideal response behavior.

To address these limitations, a classic and reliable non-glass approach is the quinhydrone electrode, in which a quinone/hydroquinone redox pair is added to the sample solution and the potential of an inert metal electrode is measured versus a reference electrode [14]. Because the redox equilibrium is proton-coupled, the electrode potential shifts with pH according to the Nernst equation [15]. Despite its conceptual simplicity, the traditional quinhydrone electrode requires dissolving the redox couple directly into the sample, which limits its applicability in systems with restricted sample volume, contamination constraints, or requirements for continuous and integrated sensing.

Recently, PCET redox systems have re-emerged as a powerful means to control electronic states in solid-state materials with high precision, exhibiting highly reproducible and Nernstian responses even at solid – liquid interfaces [16]. However, while these studies highlight the robustness of PCET-based thermodynamic control, the feasibility of implementing such equilibria in self-contained thin-film sensing devices remains largely unexplored.

In this work, we propose and evaluate a compact thin-film implementation of the quinhydrone principle by confining the benzoquinone/hydroquinone (BQ/HQ) redox couple within an ion gel and protecting the gel surface with a Nafion layer. The ion gel is placed in contact with a planar Au electrode, and the electrode potential is measured versus an Ag/AgCl reference. Nafion, a sulfonated polymer electrolyte, provides a proton-conducting pathway while acting as a barrier to reduce leaching of BQ/HQ from the device. We show that the resulting Au, ion-gel containing BQ/HQ, and Nafion stacked structure exhibits a near-Nernstian response to pH, indicating that the proton activity in the external solution is effectively transduced into a stable redox potential in a reagent-free, film-compatible format.

## Device fabrication

The BQ/HQ redox couple can be described by the two-proton-two-electron transfer reaction

(1) $$\mathrm{BQ}+2H^{+}+2e^{-}⇌\mathrm{HQ}.$$

**Figure 1.**
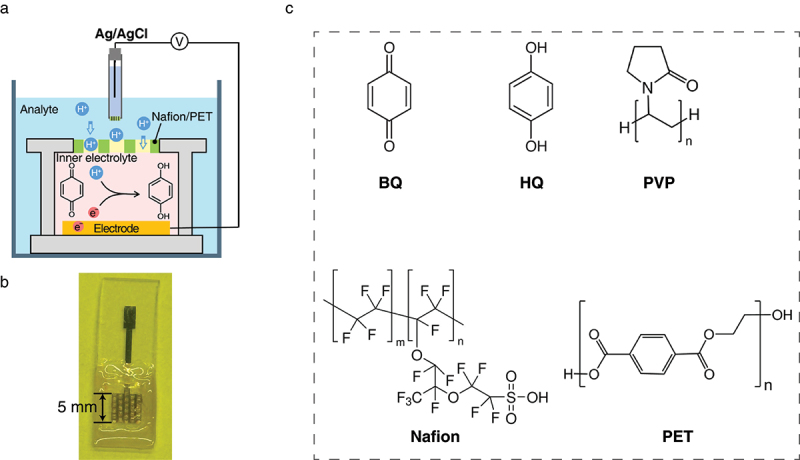
Structure and working principle of the device. (a) Schematic illustration of the device structure and working principle. (b) Photograph of the fabricated device after measurement. (c) Chemical structures of BQ, HQ, PVP, Nafion and PET.

Assuming that the activity ratio $a_{\mathrm{BQ}}/a_{\mathrm{HQ}}$ in the ion gel remains approximately constant and does not vary significantly with external pH under the present measurement conditions, the equilibrium potential versus a reference electrode follows

(2) $$E=E^{\circ^{\prime }}+\frac{\mathrm{RT}}{2F}\ln\left(\frac{a_{\mathrm{BQ}}a_{H^{+}}^{2}}{a_{\mathrm{HQ}}}\right)\approx E_{0}-\frac{2.303\mathrm{RT}}{F}\mathrm{pH},$$

where E is the electrode potential, $E^{\circ^{\prime }}$ is the formal potential of the redox couple, R is the gas constant, T is the absolute temperature, F is the Faraday constant, and $a_{\mathrm{BQ}}$, $a_{\mathrm{HQ}}$, and $a_{H^{+}}$ are the activities of benzoquinone, hydroquinone, and protons, respectively. This yields an ideal slope of −59mV/pH at 25 ${\;}^{\circ }C$. In the proposed device, Nafion provides an effective proton-activity coupling pathway between the external solution and the ion gel, allowing changes in external pH to modulate the confined BQ/HQ redox equilibrium while retaining the redox couple within the film (Figure 1a).

The thin-film sensing electrode consists of three components: (i) a planar Au working electrode, (ii) an ion gel containing dissolved BQ/HQ in direct contact with the Au surface, and (iii) a Nafion overlayer covering the gel (Figure 1b). A Nafion solution was drop-cast onto a polyethylene terephthalate (PET) film with a 0.5 mm opening to form a Nafion coating. The ion gel solution was then spin-coated onto the Nafion-coated surface. The ion gel solution was prepared from an aqueous electrolyte containing the polymeric gelling agent poly(vinylpyrrolidone) (PVP, $M_{w}\approx 10,000$, Figure 1c), with 0.1 M Na${\;}_{2}$SO${\;}_{4}$ and 5 mM of benzoquinone and hydroquinone. Finally, the assembled Nafion-coated/ion gel membrane was placed onto a planar Au electrode, with the ion-gel side contacting the Au electrode to establish electrical contact.

## Measurements of pH response

The potential difference between the fabricated BQ/HQ ion gel electrode and an Ag/AgCl reference electrode was measured while varying the pH of the external solution. If changes in the proton activity of the external solution are effectively coupled to the ion gel through Nafion, the redox potential of the confined BQ/HQ couple is expected to shift accordingly, leading to a corresponding shift in the electrode potential.

**Figure 2.**
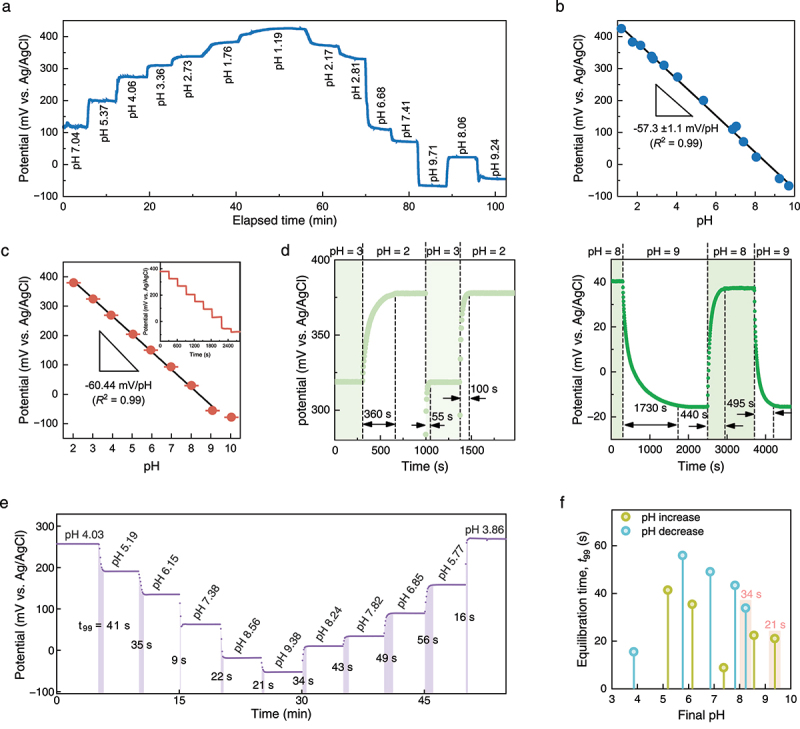
Potentiometric pH response and response kinetics of the device and bulk BQ/HQ solution. (a) Real-time potential response of the device measured versus Ag/AgCl. (b) Steady-state potential of the device as a function of pH extracted from the response shown in (a). (c) Potential of the BQ/HQ redox couple measured versus Ag/AgCl in solutions of different pH. (d) Representative transient responses of the thin-film device in the acidic and high-pH regions after prolonged equilibration. (e) Transient potential response of bulk BQ/HQ solution without Nafion or ion-gel confinement during sequential pH changes. The shaded regions indicate the transient intervals used for response-time analysis, and the values above them denote the corresponding equilibration times, $t_{99}$. (f) Equilibration time, $t_{99}$, of bulk BQ/HQ plotted against the final pH of each transition. The shaded areas highlight the transitions into and out of the high-pH region relevant to the delayed response observed in the thin-film device.

A clear pH response was observed over the range of pH 1–10 (Figure 2a). The measured slope of 57.3 mV/pH is close to the theoretical Nernstian value (Figure 2b), confirming that the observed potential change originates from the PCET process.

The observed pH response can be attributed to three key characteristics of the sensor: Nernstian behavior, response kinetics, and small drift. These aspects are discussed below. First, regarding the Nernstian response, it was not self-evident that a PCET process identical to that in aqueous solution would be observed in the ion gel environment. In aqueous solution, the BQ/HQ couple is known to exhibit pH dependence corresponding to a two-electron, two-proton process below approximately pH 10 (Figure 2c) [17]. The present results are consistent with this behavior, indicating that the same reaction mechanism governs the redox equilibrium in the gel phase.

At higher pH, a two-electron, one-proton process becomes thermodynamically favorable in aqueous media, leading to deviations from linear Nernstian behavior [18]. In fact, in our system, a deviation from linearity is observed above approximately pH 10 in aqueous conditions. In contrast, in the gel system, linearity is maintained up to pH 10. One possible origin of this difference is that the product of the two-electron, one-proton reaction is an anionic species. In a medium with a lower dielectric constant than water, such as the present ion gel, the formation of charged quinone species is energetically less favorable [19]. Therefore, the neutral two-electron, two-proton process is expected to remain dominant in the ion gel over a wider pH range compared to aqueous systems.

We performed density functional theory (DFT) calculations to further support this interpretation. Here, calculations were performed using the B3LYP functional with the 6–31++G(d,p) basis set. Solvation effects were considered using either a polarizable continuum model with a dielectric constant of ε=45, representing the PVP-based ion gel, or the solvation model based on density (SMD) with water as the solvent. Based on the calculations, upon transfer from water to the PVP-based gel environment, BQ and HQ are destabilized by 8.4 meV and 11.2 meV, respectively, indicating only minor changes in the neutral redox pair. In contrast, the charged intermediate (HQ lacking one proton) is destabilized much more strongly, by 37.6 meV. These results suggest that the gel phase not only enables the two-electron, two-proton PCET process but also improves its selectivity over an extended pH range, making it advantageous for high-pH operation.

## Response kinetics

To evaluate the response kinetics of the device, solution-exchange experiments were performed after prolonged equilibration in representative acidic and high-pH regions within the operating range (Figure 2d). The equilibration time, $t_{99}$, was defined as the time required for the device potential to reach 99% of the total potential change. The device generally responded more slowly in the high-pH region than in the acidic region. In particular, after prolonged immersion under alkaline conditions, a substantially delayed response with an equilibration time of up to 1730 s was observed, indicating that the transient response depends not only on the current external pH but also on the preceding immersion history.

To determine whether the slower and history-dependent response in the high-pH region arises from the BQ/HQ redox couple itself, we further evaluated the transient potential response of bulk BQ/HQ solution without Nafion or ion-gel confinement (Figures 2(e) and 2(f)). In this experiment, the pH of the bulk BQ/HQ solution was increased stepwise from pH 4.03 to pH 9.38 and subsequently decreased to pH 3.86. The equilibration times, $t_{99}$, ranged from 9 to 56 s for all investigated pH transitions. In particular, the transitions into and out of the high-pH region, pH 8.56 → 9.38 and pH 9.38 → 8.24, showed equilibration times of only 21 and 34 s, respectively. Thus, the bulk BQ/HQ solution did not exhibit pronounced slowing upon approaching or exceeding pH 9 and did not reproduce the strongly delayed response observed in the thin-film device after prolonged alkaline immersion.

The difference between the bulk solution and the thin-film device indicates that the slow and history-dependent response of the device is unlikely to originate predominantly from the intrinsic pH-dependent response behavior of the bulk BQ/HQ redox couple, but is more plausibly associated with slow ionic transport and equilibration processes specific to the device architecture. In particular, the fixed sulfonate sites in Nafion provide pathways for cation exchange and transport in the hydrated membrane. Because the phosphate-buffer-based external solutions used for the device measurements contain Na${\;}^{+}$, competitive exchange and transport of H${\;}^{+}$ and Na${\;}^{+}$ within Nafion may retard the establishment of effective proton-activity coupling between the external solution and the ion gel, thereby prolonging the device response [20,21]. Ion redistribution within the confined ion-gel environment may also contribute to the dependence of the response on the preceding immersion state.

Accordingly, the slow and history-dependent response observed in the high-pH region is consistent with contributions from coupled cation-exchange/transport processes in the Nafion layer and slow re-equilibration within the confined ion gel. Nevertheless, the device ultimately converges to a well-defined equilibrium potential determined by the pH of the external solution.

## Long-term stability and drift

Regarding drift, the present measurements show only minor deviation from the ideal pH-dependent potential change, indicating good stability of the sensing system. Drift is an inherent issue in electrochemical sensors, including Ag/AgCl reference electrodes, typically due to changes in the internal electrolyte concentration [22]. In the present device, Nafion suppresses the leaching of ion gel components; however, changes in the BQ/HQ ratio caused by slow leakage, reaction with dissolved oxygen, or parasitic electrochemical processes under measurement current could potentially induce potential drift. To evaluate these effects, long-term stability tests were conducted, as described below.

**Figure 3.**
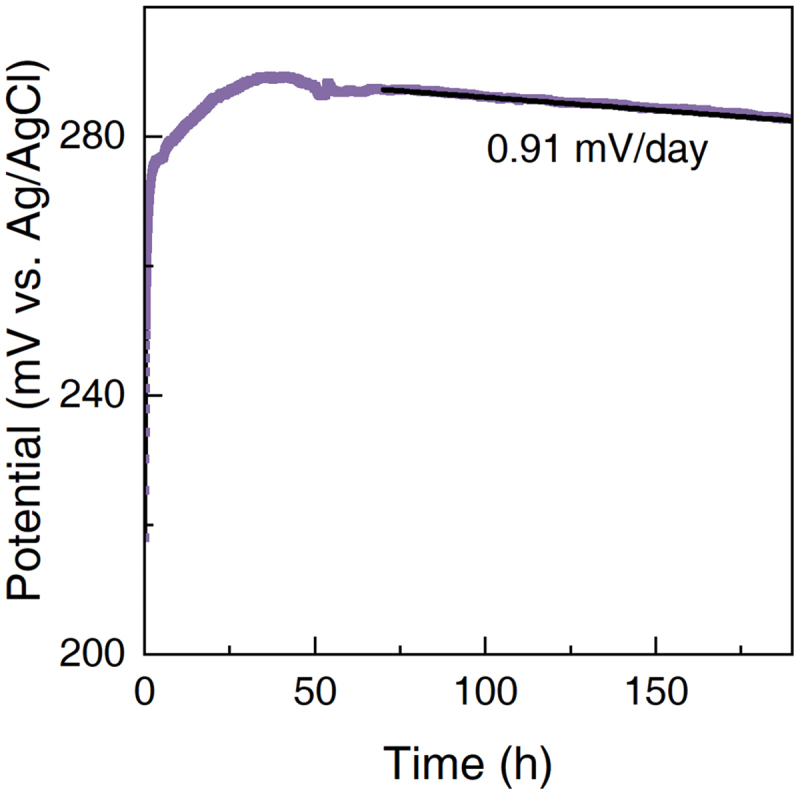
Long-term stability of the device.

Long-term drift of the sensor was evaluated in a pH 3.5 buffer solution under dark conditions at 25 ${\;}^{\circ }C$. The results are shown in Figure 3. After an initial fluctuation period, the sensor exhibited a gradual and stable drift. Beyond 75 h, the drift rate was as low as 0.9 mV/day. This drift corresponds to an estimated potential shift of approximately 6.3 mV over one week. Considering the measured pH sensitivity of 57.3 mV/pH, this translates to a pH deviation of only 0.11 units after one week of operation.

Film-type sensors can be fabricated in large quantities from a single substrate using simple processing steps. The present results therefore indicate that even a straightforwardly fabricated film sensor can maintain practical accuracy for at least one week of continuous use. For comparison, conventional glass pH electrodes typically exhibit drift on the order of 0.5–2 mV/day under laboratory conditions, depending on the electrolyte stability and reference junction characteristics. The drift observed in the present device is thus of the same order of magnitude as that of widely used laboratory-grade pH electrodes.

Considering these points, part of the observed potential shift may originate from the Ag/AgCl reference electrode rather than from the BQ/HQ redox system itself. A detailed investigation of the drift mechanism, including possible contributions from the reference electrode, redox composition changes, and interfacial processes, remains an important subject for future study.

## Discussion

We presented a thin-film pH sensor that confines a BQ/HQ redox couple within an ion gel in contact with an Au electrode and employs a Nafion overlayer to enable proton transport while suppressing redox-species leaching. The open-circuit potential measured versus Ag/AgCl exhibits a Nernstian dependence on pH, indicating successful implementation of the quinhydrone electrode concept in a compact and reagent-free format. This approach provides a promising alternative to glass electrodes for miniaturized and integrated pH sensing.

The present architecture enables the deployment of a large number of thin-film sensing electrodes through simple fabrication processes, allowing dense spatial distribution of sensing elements. Because these electrodes can be operated against a single reference electrode, this configuration naturally enables multi-point pH mapping across a given system. Such capability is particularly advantageous for monitoring spatially heterogeneous biochemical environments and dynamic processes. While the reference electrode remains a comparatively bulky component, the ability to integrate dense arrays of flexible sensing electrodes significantly expands the scope of electrochemical measurements without requiring full miniaturization of all device components.

Considering the possibility of thin-film integration of Ag/AgCl reference electrodes [23–25], the present architecture may enable the realization of a fully thin-film pH sensor platform. Furthermore, integration with semiconductor materials or transistor-based architectures could allow amplification and signal processing within the same device structure. Such devices could serve as pH transducers in miniaturized electrochemical systems, including microfluidic platforms, wearable patches, and distributed sensor arrays.

## Method

Potentiometric characterization was carried out using a DMM2000 digital multimeter (Keithley) with an Ag/AgCl reference electrode. For real-time measurements, the device was immersed in phosphate buffer solution, and the solution pH was adjusted by the addition of phosphoric acid (H${\;}_{3}$PO${\;}_{4}$) and sodium hydroxide (NaOH).

The pH dependence of the BQ/HQ redox potential in solution was evaluated using aqueous solutions containing 5 mM benzoquinone (BQ) and 5 mM hydroquinone (HQ). Solutions with different pH values were prepared using sulfuric acid (H${\;}_{2}$SO${\;}_{4}$), sodium sulfate (Na${\;}_{2}$SO${\;}_{4}$), and NaOH.

To fabricate a device, a Nafion solution was drop-cast onto a polyethylene terephthalate (PET) film with a 0.5 mm opening to form a Nafion coating. The ion gel precursor was then spin-coated onto the Nafion-coated surface. The ion gel precursor was prepared from an aqueous electrolyte containing the polymeric gelling agent poly(vinylpyrrolidone) (PVP, $M_{w}\approx 10,000$), 0.1 M Na${\;}_{2}$SO${\;}_{4}$, and 5 mM each of benzoquinone and hydroquinone. The resulting Nafion/ion-gel membrane was placed onto a planar Au electrode, with the ion-gel side contacting the Au electrode to establish electrical contact.

For transient response measurements, phosphate buffer solutions of predefined pH values were prepared separately. After each measurement step, both the device and the Ag/AgCl reference electrode were rinsed, followed by immersion in the next solution. The potential was then recorded continuously as a function of time.

For the response-kinetics measurement of bulk BQ/HQ, transient potential responses were recorded for an aqueous solution containing 5 mM BQ, 5 mM HQ, and 0.1 M Na${\;}_{2}$SO${\;}_{4}$. The potential was measured versus an Ag/AgCl reference electrode while the pH of the same solution was sequentially increased from pH 4.03 to pH 9.38 by adding NaOH and subsequently decreased to pH 3.86 by adding H${\;}_{2}$SO${\;}_{4}$. The equilibration time, $t_{99}$, was defined as the time required for the potential to reach 99% of the total potential change after each pH transition, consistent with the criterion used for the thin-film device.

For the long-term stability measurement, the device was immersed in a pH 3.5 buffer solution, and its potential versus an Ag/AgCl reference electrode was continuously recorded under dark conditions at 25 ${\;}^{\circ }C$. The pH of the external solution was measured before and after the stability test using a calibrated pH meter. The drift rate was evaluated by linear fitting of the potential change after 75 h.

## Data Availability

The data that supports the findings of this study are available within the article.
